# Comparative genomic analysis between *Corynebacterium pseudotuberculosis* strains isolated from buffalo

**DOI:** 10.1371/journal.pone.0176347

**Published:** 2017-04-26

**Authors:** Marcus Vinicius Canário Viana, Henrique Figueiredo, Rommel Ramos, Luis Carlos Guimarães, Felipe Luiz Pereira, Fernanda Alves Dorella, Salah Abdel Karim Selim, Mohammad Salaheldean, Artur Silva, Alice R. Wattam, Vasco Azevedo

**Affiliations:** 1 Departament of General Biology, Federal University of Minas Gerais, Belo Horizonte, Minas Gerais, Brazil; 2 Biocomplexity Institute of Virginia Tech, Virginia Tech, Blacksburg, Virginia, United States of America; 3 AQUACEN, National Reference Laboratory for Aquatic Animal Diseases, Ministry of Fisheries and Aquaculture, Federal University of Minas Gerais, Belo Horizonte, Minas Gerais, Brazil; 4 Center of Genomic and System Biology, Federal University of Pará, Belém, Pará, Brazil; 5 Department of Microbiology, Faculty of Veterinary Medicine, Cairo University, Giza, Egypt; Defense Threat Reduction Agency, UNITED STATES

## Abstract

*Corynebacterium pseudotuberculosis* is a Gram-positive, pleomorphic, facultative intracellular pathogen that causes Oedematous Skin Disease (OSD) in buffalo. To better understand the pathogenic mechanisms of OSD, we performed a comparative genomic analysis of 11 strains of *C*. *pseudotuberculosis* isolated from different buffalo found to be infected in Egypt during an outbreak that occurred in 2008. Sixteen previously described pathogenicity islands (PiCp) were present in all of the new buffalo strains, but one of them, PiCp12, had an insertion that contained both a corynephage and a diphtheria toxin gene, both of which may play a role in the adaptation of *C*. *pseudotuberculosis* to this new host. Synteny analysis showed variations in the site of insertion of the corynephage during the same outbreak. A gene functional comparison showed the presence of a nitrate reductase operon that included genes involved in molybdenum cofactor biosynthesis, which is necessary for a positive nitrate reductase phenotype and is a possible adaptation for intracellular survival. Genomes from the buffalo strains also had fusions in minor pilin genes in the *spaA* and *spaD* gene cluster (*spaCX* and *spaYEF*), which could suggest either an adaptation to this particular host, or mutation events in the immediate ancestor before this particular epidemic. A phylogenomic analysis confirmed a clear separation between the Ovis and Equi biovars, but also showed what appears to be a clustering by host species within the Equi strains.

## Introduction

*Corynebacterium*, *Mycobacterium*, *Nocardia*, and *Rhodococcus*, collectively known as the CMNR group, are all members of the order Corynebacteriales. This group contains species of medical, veterinary and biotechnological importance with shared common characteristics that include a high GC content and a cell wall composed mainly of peptidoglycans, arabinogalactans and mycolic acids [1]. *Corynebacterium* species are Gram-positive, rod-shaped, non-motile and non-spore forming bacteria [2], and include both pathogens like *C*. *diphtheria*, *C*. *jeikeium* and *C*. *pseudotuberculosis*, and non-pathogenic strains like *C*. *glutamicum* [3]. *Corynebacterium pseudotuberculosis* are facultative intracellular pathogens that are divided into two distinct biovars, each of which can infect a range of mammalian hosts. Nitrate reductase activity seems to correlate with the type of host that they infect, and thus the biovar that each strain belongs to. Isolates belonging to the Ovis biovar infect sheep and goats, and they are nitrate reductase negative. Members of the Equi biovar infect larger hosts like the horses, cattle, and buffalo [4], and they are have positive nitrate reductase activity [5,6]. The separation into two biovars is also supported by molecular markers [6,7] and by distinct differences in gene content that has observed across genomes [8].

*C*. *pseudotuberculosis* causes different diseases and symptoms in these various hosts. Infection in goats and sheep results in a manifestation called Caseous Lymphadenitis [9]. Infected cattle have ulcerative granulomatous lesions and mastitis [10,11], while horses are diagnosed with ulcerative lymphangitis, or with pigeon fever, so named due to a swelling in the chest of infected animals [12]. Oedematous Skin Disease (OSD) is the manifestation seen in buffalo. OSD is characterized by an extensive cutaneous, oedematous swelling in the dewlap, whole hind or forelimbs, and belly [13]. The disease causes economic loss due to reduced milk and meat production as well as reduced work efficiency of the animals. Moreover, the treatment is expensive and can last for months [13,14]. OSD is endemic in regions of lower Egypt and the areas surrounding Cairo, with sporadic cases throughout the year and periodic epidemics seen in the summer [13]. Unlike some of the other hosts that are infected by *C*. *pseudotuberculosis*, there is no documented transmission directly between infected buffalo. Instead, it has been suggested that insects are involved with transmitting the disease between these animals. Specifically, biting flies of the genus *Hippobosca* have been associated with transmission, the suggestion being that they inject the bacteria intradermally with each bite [15]. Further proof of involvement is the close correlation between the breeding season of these blood-sucking flies with outbreaks of OSD in buffalo [13].

All strains that have been isolated in Egypt from infected buffalo belonged to the Equi biovar, and all those sequenced genomes contain a gene that produces the diphtheria toxin [16]. This toxin is found in other *Corynebacterium* species, but within *C*. *pseudotuberculosis* it has only been found in isolates from buffalo to date. It is unclear if the presence of this gene indicates an adaptation that allows it to specifically infect buffalo, or if it is present due to a horizontal transfer event that occurred recently and spread across the specific geographic region where the animals were found [8].

To fully characterize these isolates and determine additional genomic features that not only distinguish them, but might explain the pathogenic mechanisms of OSD, a comparative analysis of 11 strains of *C*. *pseudotuberculosis* isolated from different buffalo in Egypt was conducted. These genomes were compared to other *C*. *pseudotuberculosis* strains from both Ovis and Equi biovars. Here we describe those differences, explore the species-wide pangenome, and define the differences related to not only the biovars, but also to the hosts they have been isolated from.

## Materials and methods

### Strains isolation

Each of the 11 *C*. *pseudotuberculosis* strains were isolated from individual water buffalo (*Bubalus bubalis*) in Egypt during an outbreak of OSD that occurred during the summer of 2008 (Table 1). The infected animals were all located in separate farms either in the Menofia (located 89 km north of Cairo) or El-Fayoum (50 km west Cairo) regions. The samples were collected following permission from the owners of the individual animals. No additional permission was required as Egyptian buffalo are not protected, and are considered to be domesticated livestock. Samples were collected from internal lesions that were located within the brisket, fore or hind limbs of these animals. The samples were plated on selective media (Brain Heart Agar with Fosfomycin). A single colony from each of the samples was selected for species identification, and identification of *C*. *pseudotuberculosis* was determined by API Coryne (BioMerieux, France) [17] according to the manufacturer’s instructions. An aliquot of each sample was lyophilized and sent to the Laboratory of Cellular and Molecular Genetics in Brazil where they were grown again, and a second confirmation showed them to be *C*. *pseudotuberculosis*. Each strain was demonstrated to produce diphtheria toxin by both API Coryne System and multiplex PCR tests [18]. Colonies from these pure cultures were plated on agar, and one colony from each of these was randomly selected for sequencing.

**Table 1:** Strains of *Corynebacterium pseudotuberculosis* isolated from buffalo diagnosed with Oedematous Skin Disease in Egypt during the summer of 2008.

Strain	Genome size (bp)	Mean coverage depth	*De novo* assembler	CDS	tRNA	rRNA	Accession number
31 [19]	2,402,956	550x	MIRA 4.0.2; SPADES 3.1.1	2248	47	12	CP003421.3
32	2,403,533	56.61x	Newbler 2.9	2272	52	12	CP015183
33	2,403,550	104.11x	Newbler 2.9	2281	52	12	CP015184
34	2,403,454	58.94x	MIRA 3.9.18	2286	49	12	CP015192
35	2,403,502	112.68x	Newbler 2.9	2276	52	12	CP015185
36	2,403,412	172.02x	SPAdes 3.6.0	2279	49	12	CP015186
38	2,403,515	160.27x	SPAdes 3.6.0	2281	49	12	CP015187
39	2,403,579	241.79x	SPAdes 3.6.0	2281	49	12	CP015188
43	2,365,075	218.07x	Newbler 2.9	2218	51	12	CP015189
46	2,366,565	249.57x	SPAdes 3.6.0	2218	48	12	CP015190
48	2,403,301	306.54x	SPAdes 3.6.0	2281	49	12	CP015191

### Genome sequencing and assembly

**Fig 1:**
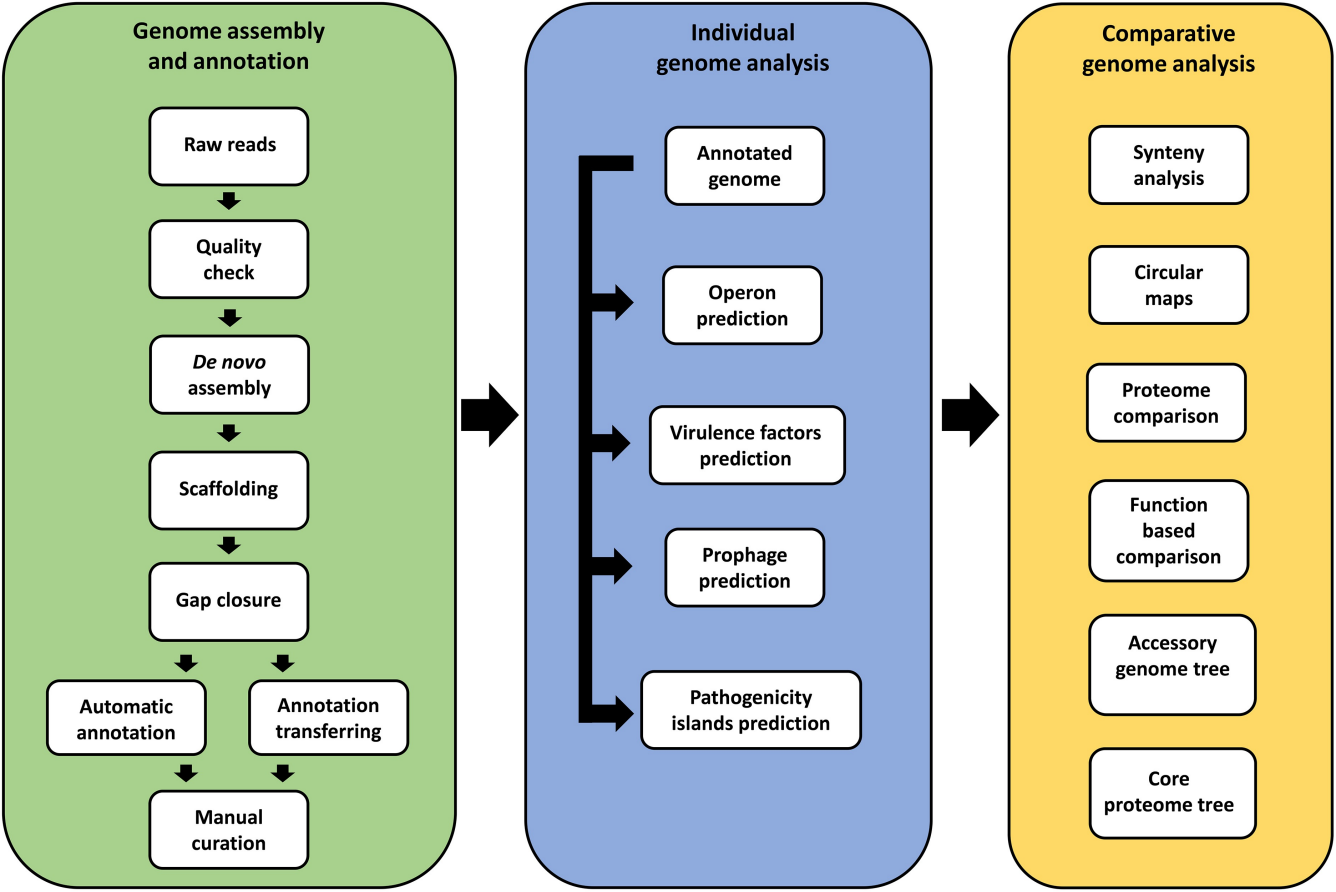
Workflow of the genome assembly and annotation of 11 *Corynebacterium pseudotuberculosis* strains isolated from buffalo, and comparative genomics analysis with other strains.

The *in silico* analysis workflow is presented in Fig 1. Sequencing, assembly and annotation of individual genomes was conducted at one of three laboratories. Two of these (Laboratory of Cellular and Molecular Genetics and the National Reference Laboratory for Aquatic Animal Diseases) are part of the Federal University of Minas Gerais, Belo Horizonte, Minas Gerais, Brazil. The third (Center of Genomic and System Biology) is located at the Federal University of Pará in Belém, Pará, Brazil. All genomes were sequenced by Ion Personal Genome Machine (PGM) with the Ion 318^TM^ chip, using Ion PGM Template OT2 400 Kit and Ion PGM Hi-Q Sequencing Kit. The quality of the reads for each genome was examined using FastQC 0.10.1 (http://www.bioinformatics.babraham.ac.uk/projects/fastqc/).

Simba 1.2.1 [20] was used to assemble the genomes, unite them into a single scaffold, and establish the start of the chromosome. Simba includes three different assembly packages: SPAdes 3.6.0 [21]; MIRA 3.9.18 (http://sourceforge.net/projects/mira-assembler/); and Newbler 2.9 (http://swes.cals.arizona.edu/maier_lab/kartchner/documentation/index.php/home/docs/newbler). A “best” assembly was selected for each isolate based on having the highest N50, the smallest number of contigs, and higher values of maximum and minimum contigs size [22]. The contigs of this “best” assembly were then united into a single scaffold using CONTIGuator [23], with *C*. *pseudotuberculosis* 31 (CP003421.2) [19] used as a reference. The overlap between contigs was verified by BLASTN, and an in-house script was used to establish the beginning of the chromosome at the *dnaA* gene. The gaps were closed using CLC Genomics Workbench 6.5 (http://www.clcbio.com/products/clc-main-workbench/) in the following way. First, the reads were mapped to the reference genome to establish a consensus sequence. Next, the sequences of the flanking regions around each gap were identified in the reference genome. Then, the consensus sequence that had been generated from the reads was used to fill the gap when it was inserted between the flanking sequences.

### Genome annotation

All genomes, including strain 31 (CP003421.2) [24], were annotated consistently using the RASTtk (Rapid Annotation Using Subsystem Technology) [25] annotation service in PATRIC (Pathosystems Resource Integration Center) [26]. To verify the authenticity of the identified frameshifts, a specific curation process that involved manual examination of potential frameshifts was performed. Insertion/deletion (indel) errors are associated to a certain extent with any sequencing platform, and those associated with homopolymers in the Ion Torrent sequencing platform are well defined [27]. Our curation process involved the following steps. First, a manually curated annotation of the reference genome was transferred to each of the other 10 genomes using an in-house script to identify the pseudogenes. Frameshifts were examined in Artemis v16.0.0 [28]. To identify the frameshift location and see if the mutation was conserved across other genomes, the genes with potential frameshifts were compared to other complete genes by BLASTN against the NR database at NCBI (National Center for Biotechnology Information). Second, we checked for indels among the reads at the location of the frameshift by mapping them against the assembled genome using the CLC Genomics Workbench v6.5. When an examination of the reads showed that an identified frameshift was a sequencing artifact, the sequence was adjusted. The new sequence, translated into a protein sequence, was verified by BLASTP against the Uniprot database [29]. The genome with the corrected sequences, which included the fixed frameshifts, was then re-annotated using RASTtk.

### Genome plasticity

Pathogenicity islands (PAIs) were predicted by GIPSy [30]. All 11 genomes were individually compared to the *C*. *glutamicum* ATCC1302 genome (NC_006958.1), a non-pathogenic strain. DoubleACT v2 (http://www.hpa-bioinfotools.org.uk/pise/double_act.html) was used to align the pairs of genomes, and ACT (Artemis Comparison Tool) v13.0.0 [28] was used to visualize the alignments and check if the sequence of a predicted island in one genome was present but not annotated in the second genome. Comparison maps of the genomes were generated using BRIG (Blast Ring Image Generator) v0.95 [31].

PHAST, which predicts and annotates prophages from raw DNA sequence data or Genbank files, was used to predict prophages in the genomes by BLASTing against the NCBI and the prophage databases [32]. Syntenic and unique regions were both verified in the genomes by the progressiveMauve algorithm [33].

### Phylogenomics

Gegenees v2.2.1 [34] was used to create a matrix of similarity across the genomes. This matrix was exported as a nexus file and used to generate a phylogenomic tree using Splitstree v4.14.2 [35] with the UPGMA method.

The PEPR (Phylogenomic Estimation with Progressive Refinement) program (https://github.com/enordber/pepr.git) was used to generate a phylogenomic tree with the publicly available and complete *C*. *pseudotuberculosis* genomes, and also included the new buffalo isolates. This is an automated system for generation of phylogenomic trees from amino acid sequences by a maximum likelihood algorithm. It identifies the common orthologs among all genomes, filters out genes that have been transferred horizontally, aligns and concatenates sequences, and generates a tree. Subtrees with low bootstrap values are refined by subsequent steps of addition genes that are shared across smaller clusters. This method is appropriated for uneven sampling in databases, where taxons of interest have a denser sampling. The resulting Newick tree file was visualized using Mega 6 [36].

### Pangenomics

We compared the 11 buffalo isolates with the 33 other genomes publicly available in NCBI (Table 2). The Protein Family Sorter [26] was used to examine the pan-, core- and accessory genomes using the *Corynebacterium* genus-specific protein families (PLfams) that are available in PATRIC [37]. In addition, PATRIC’s Proteome Comparison tool (https://www.patricbrc.org/app/SeqComparison), an adaptation of RAST’s Sequence Based Comparison Tool [38], was used to generate a matrix of the bidirectional BLASTP hits across the proteins annotated in the genomes, with each genome used in a separate comparison as the reference genome to which the other 10 were compared. RAST’s Function based Comparison tool (http://rast.nmpdr.org/seedviewer.cgi) was used to assess similarities and differences in the presence of functional roles among the genomes. FgenesB (http://www.softberry.com/berry.phtml?topic=fgenesb) was used to identify operons in regions of interest.

**Table 2:** List of the 33 strains of *Corynebacterium pseudotuberculosis* used in this study that were isolated from hosts other than buffalo.

Strain	Biovar	Host	Country	Accession number
Cp162	Equi	Camel	UK	CP003652.1
262	Equi	Cow	Belgium	CP012022.1
258	Equi	Horse	Belgium	CP003540.2
E19	Equi	Horse	Chile	CP012136.1
CIP52.97	Equi	Horse	Kenya	CP003061.1
1/06-A	Equi	Horse	USA	CP003082.1
316	Equi	Horse	USA	CP003077.1
MB11	Equi	Horse	USA	CP013260.1
MB14	Equi	Horse	USA	CP013261.1
MB30	Equi	Horse	USA	CP013262.1
MB66	Equi	Horse	USA	CP013263.1
29156	Ovis	Cow	Israel	CP010795.1
I19	Ovis	Cow	Israel	CP002251.1
1002B	Ovis	Goat	Brazil	CP012837.1
VD57	Ovis	Goat	Brazil	CP009927.1
CS_10	Ovis	Goat	Norway	CP008923.1
Ft_2193/67	Ovis	Goat	Norway	CP008924.1
PO222/4-1	Ovis	Goat	Portugal	CP013698.1
PO269-5	Ovis	Goat	Portugal	CP012695.1
226	Ovis	Goat	USA	CP010889.1
FRC41	Ovis	Human	France	CP002097.1
48252	Ovis	Human	Norway	CP008922.1
267	Ovis	Llama	USA	CP003407.1
N1	Ovis	Sheep	Equatorial Guinea	CP013146.1
PAT10	Ovis	Sheep	Argentina	CP002924.1
C231	Ovis	Sheep	Australia	CP001829.1
42/02-A	Ovis	Sheep	Australia	CP003062.1
12C	Ovis	Sheep	Brazil	CP011474.1
PA01	Ovis	Sheep	Brazil	CP013327.1
E56	Ovis	Sheep	Egypt	CP013699.1
MEX25	Ovis	Sheep	Mexico	CP013697.1
3/99-5	Ovis	Sheep	Scotland	CP003152.1
P54B96	Ovis	Wildebeest	South Africa	CP003385.1

### Specialty genes search

PATRIC’s Specialty Genes Search tool [39] was used to identify some of the virulence genes that were found in the 11 genomes. This tool BLASTs all genes in the annotated genome against a virulence factor database that includes VFDB [40] and manually curated virulence genes [39].

## Results and discussion

### Genome sequencing, assembly and annotation

All the 10 new genomes were closed, with all reads united into a single chromosome. For each genome, the theoretical mean coverage depth varied between 56.72 and 307.02x, while mean coverage depth genome varied between 56.61 and 306.54x. The 11 strains (including strain 31) have a genome size of approximately 2.4 Mb, a GC content that ranges between 52.07 and 52.1%, 2218 to 2,286 coding sequences (CDSs), 44 to 52 tRNA and 12 rRNA genes (Table 1). These values are within the ranges seen among other strains of *C*. *pseudotuberculosis*, which is considered to be more clonal compared to its closest relatives *C*. *diphtheriae* and *C*. *ulcerans* [8].

### Genome plasticity

Genome plasticity analysis can identify areas of the genome, known as PAIs, which have been acquired by horizontal transfer and frequently include virulence genes [30]. Sixteen PAIs (PiCp1-16) were previously described in *C*. *pseudotuberculosis* and are shared across the biovars [8,41–43]. The variability in the size, gene content and deletion patterns in these islands explain most of the genomic differences seen between the two biovars. The content of sixteen PAIs are highly conserved in all the genomes belonging to Ovis, while biovar Equi has a greater variability, specifically with regard to deletions within the pilus genes [8].

**Fig 2:**
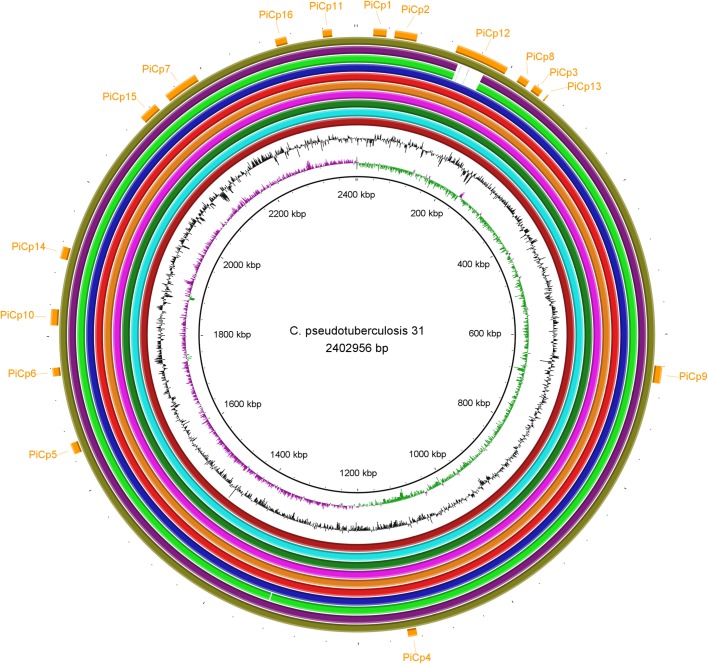
A circular genomic map that compares 11 *Corynebacterium pseudotuberculosis* strains isolated from Egyptian buffalo. The rings, from the inner to outer circle, are: strain 31, GC skew, GC content, strains 32, 33, 34, 35, 36, 38, 39, 43, 46, and 48, and pathogenicity islands.

These same sixteen PAIs were found in all the genomes isolated from buffalo (Fig 2). Interestingly, we discovered three regions that were missing in the second assembly of strain 31 (CP003421.2, re-sequenced by Ion PGM) when it was compared to other buffalo strains, but found that these regions were present in the first assembly of the same genome (CP003421.1, sequenced by Solid v3) (S1 Fig, S1 File, S1 Table). To fix the second assembly, we mapped the reads of the Ion PGM to the first assembly to verify whether those regions were represented within the reads, and to generate a consensus sequence. The second assembly was updated to include these regions, verified using an optical map, and deposited at GenBank under the accession number CP003421.3.

**Fig 3:**
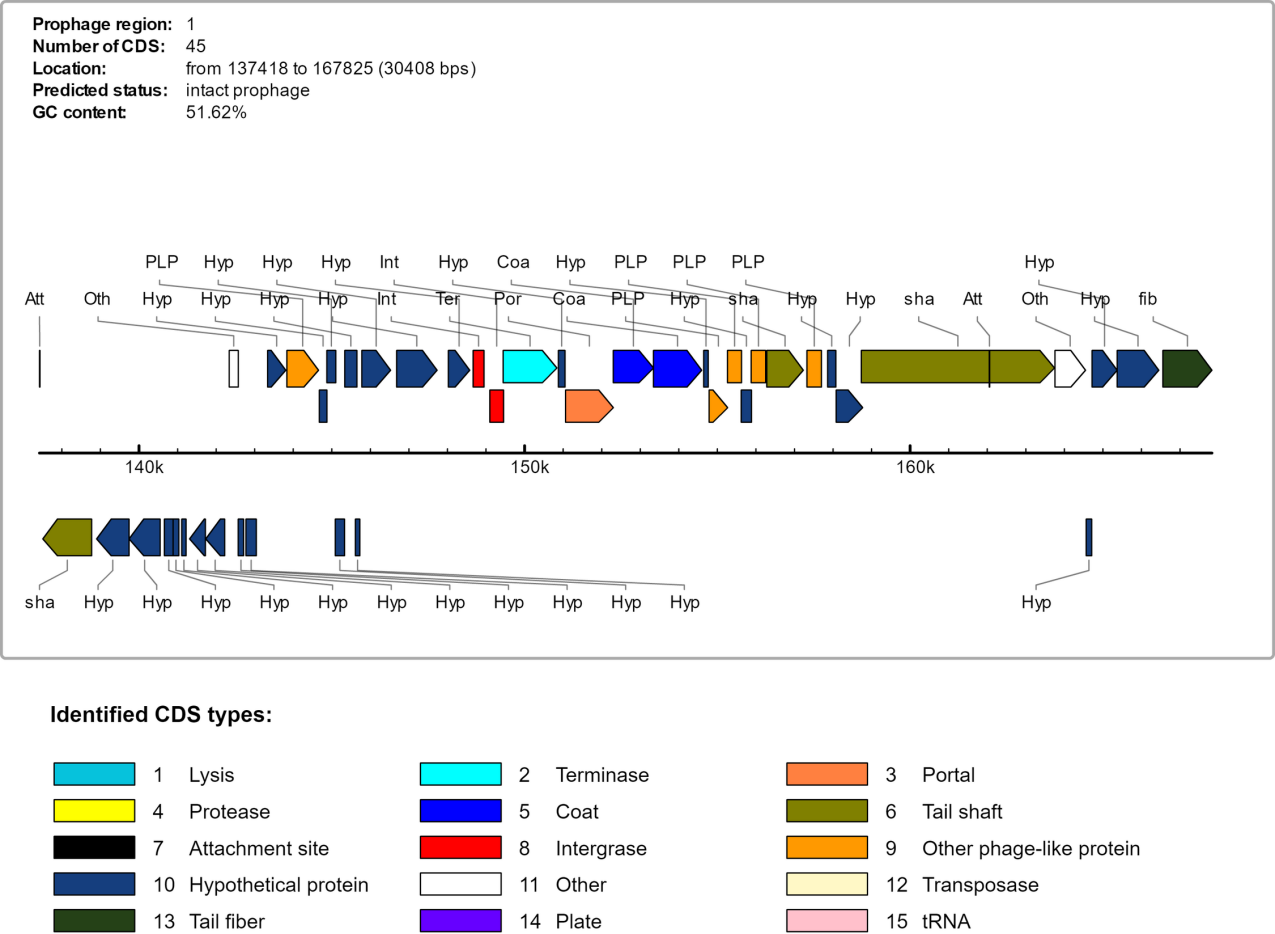
Gene content of the intact prophage predicted by PHAST. The prophage is inserted in pathogenicity island PiCp12 of the strains isolated from buffalo.

The Ovis and Equi strains share a genomic island known as PiCp12. All the buffalo isolates except strains 43 and 46 have a large insertion in PiCp12 that the other isolates from other hosts lack. This insertion is 36.6 Kb in length and includes 48 CDS and which are flanked by tRNA-Arg genes (Table 3). This insertion includes an intact β-corynephage, a prophage that was predicted by PHAST and estimated to be 30.4Kb and contains 39 CDSs (Fig 3) (Table 3, CDSs 3 to 41). A search of the NCBI database showed that the 36.6 Kb insertion sequence is unique and probably a new corynephage. This insertion also includes a tyrosine integrase gene, which codes for an enzyme known for site specific recombination [44]. tRNA-Arg genes, known as integration sites of β-corynephages [45], flank this region. This provides a possible explanation for the insertion of this unique 36.6 Kb region inside of the PiCp12 genomic island.

**Table 3:** Gene content of pathogenicity island PiCp12, a 36.6 Kb insertion sequence found in the buffalo isolates.

CDS	Position	Strand	Product	Refseq locus tag
1	136439..137053	+	Hypothetical protein	CP31_RS00695
2	137233..137475	+	Hypothetical protein	CP31_RS00700
	137418..137430	+	attL GCTAAAAAGGGGC	CP31_RS00715
3	137506..138771	-	PHAGE_Mycoba_Ariel_NC_028876: tyrosine integrase; phage(gi971751237)	CP31_RS00705
4	138901..139740	-	Hypothetical protein	CP31_RS00710
5	139742..140548	-	Hypothetical protein	CP31_RS00715
6	140658..140882	-	PHAGE_Rhodoc_REQ2_NC_016652: Hypothetical protein; phage(gi372449852)	CP31_RS00720
7	140885..141031	-	Hypothetical protein	None
8	141106..141219	-	Hypothetical protein	None
9	141313..141720	-	No significant database matches	CP31_RS00725
10	141730..141981	-	No significant database matches	CP31_RS0073
11	142339..142575	+	Transcriptional regulator	CP31_RS00735
12	142624..142884	+	Hypothetical protein	None
13	143334..143807	+	Hypothetical protein	CP31_RS00745
14	143834..144655	+	PHAGE_Bacter_Lily_NC_028841: antirepressor; phage(gi971748300)	CP31_RS00750
15	144870..145103	+	Hypothetical protein	CP31_RS00760
16	145127..145351	+	Hypothetical protein	CP31_RS00765
17	145612..145725	-	Hypothetical protein	None
18	145779..146525	+	Hypothetical protein	CP31_RS00775
19	146683..147729	+	Hypothetical protein	CP31_RS00780
20	148215..148577	+	No significant database matches	CP31_RS00785
21	148665..148946	+	PHAGE_Coryne_BFK20_NC_009799: gp55, HNH endonuclease; phage(gi157168428)	None
22	149063..149455	+	Hypothetical protein	CP31_RS00790
23	149445..150839	+	PHAGE_Coryne_BFK20_NC_009799: gp2, terminase; phage(gi157168375)	CP31_RS00795
24	150875..151048	+	Hypothetical protein	CP31_RS00795
25	151061..152302	+	PHAGE_Coryne_BFK20_NC_009799: gp3, phage portal protein; phage(gi157168376)	CP31_RS00800
26	152299..153345	+	PHAGE_Coryne_BFK20_NC_009799: gp5, head maturation protease; phage(gi157168378)	CP31_RS00805
27	153342..154592	+	PHAGE_Coryne_BFK20_NC_009799: gp6, major capsid protein; phage(gi157168379)	CP31_RS00810
28	154592..154762	+	No significant database matches	None
29	154785..155267	+	PHAGE_Coryne_BFK20_NC_009799: gp8; phage(gi157168381)	CP31_RS00815
30	155264..155626	+	PHAGE_Coryne_BFK20_NC_009799: gp9; phage(gi157168382)	CP31_RS00820
31	155619..155885	+	PHAGE_Coryne_BFK20_NC_009799: gp10; phage(gi157168383)	CP31_RS00825
32	155875..156252	+	PHAGE_Coryne_BFK20_NC_009799: gp11; phage(gi157168384)	CP31_RS00830
33	156281..157228	+	PHAGE_Coryne_BFK20_NC_009799: gp12, major tail protein; phage(gi157168385)	CP31_RS00835
34	157323..157700	+	PHAGE_Coryne_BFK20_NC_009799: gp13; phage(gi157168386)	CP31_RS00840
35	157862..158071	+	No significant database matches	CP31_RS00845
36	158081..158770	+	PHAGE_Coryne_BFK20_NC_009799: gp15, minor tail protein; phage(gi157168388)	CP31_RS00850
37	158733..163745	+	PHAGE_Coryne_P1201_NC_009816: putative tail measure protein; phage(gi157310951)	CP31_RS00850
	162051..162063	+	attR GCTAAAAAGGGGC	None
38	163755..164546	+	Immunity-specific protein Beta201	CP31_RS00855
39	164587..165369	+	Immunity-specific protein Beta286	CP31_RS00860
40	165370..166449	+	Immunity-specific protein Beta371	CP31_RS00865
41	166449..167825	+	PHAGE_Salmon_Fels_1_NC_010391: putative bacteriophage tail fiber protein; Lambda gpN homolog; phage(gi169257204)	CP31_RS00870
42	167829..168005	+	Hypothetical protein	None
43	168307..168861	+	Hypothetical protein	CP31_RS00875
44	168969..169706	+	Teichoic acid phosphorylcholine esterase/choline binding protein E (cbpE)	CP31_RS00880
45	169703..169945	+	Hypothetical protein	CP31_RS00885
46	169948..170412	+	Putative membrane protein	CP31_RS00890
47	170409..170747	+	Putative secreted protein	CP31_RS00895
48	171092..172774	+	Diphtheria toxin	CP31_RS00900

The sequence that is most similar to this new corynephage is BFK20 (NC_009799), a non-toxigenic dsDNA virus that infects *Brevibacterium flavum*. BFK20 has structural and lytic genes closely related to known phages found in the Corynebacterinae and to *C*. *diphtheriae* prophages [46]. The sequence of the new β-corynephage of strain 31 was deposited in Genbank under accession number KY566218. Additional accession numbers for the sequences of all of the buffalo strains are provided in S2 Table.

**Fig 4:**
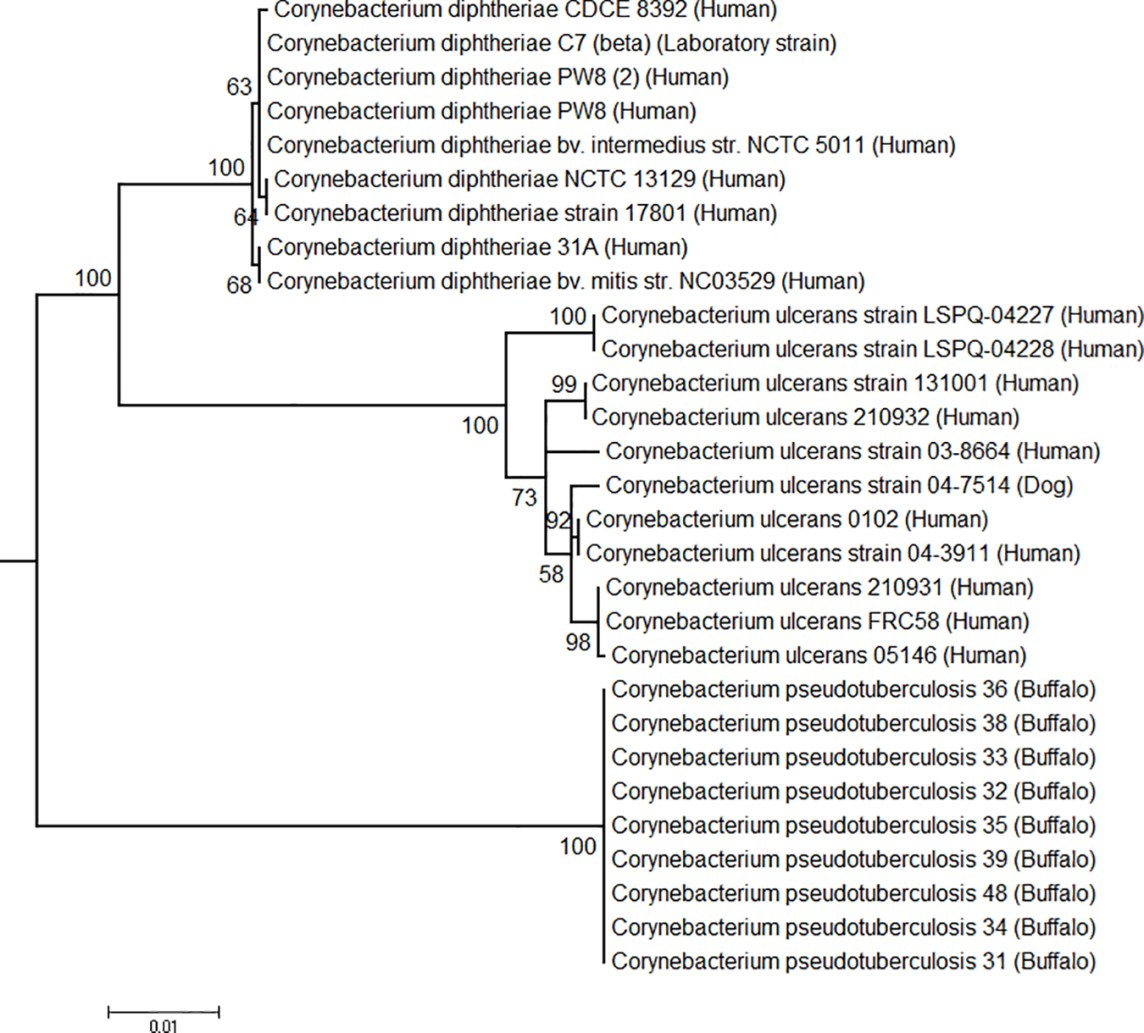
Phylogeny of diphtheria toxin gene (*tox*) from *Corynebacterium pseudotuberculosis*, *C*. *diphtheriae* and *C*. *ulcerans* inferred by using the maximum likelihood method based on the Tamura 3-parameter model, on Mega v6.

The 3’ end of the 36.6 Kb insertion into the PiCp12 island includes a gene of particular interest that produces the diphtheria toxin. This gene is only found in *C*. *pseudotuberculosis* strains that were isolated from buffalo, and is found 3,266 bp from the end of the predicted prophage in our isolates. The sequence is identical in all isolates, and differs from the toxin genes that are found in *C*. *diphtheria* and *C*. *ulcerans* (Fig 4). Maximescu et al. [47] have reported the presence of a diphtheria toxin from two separate strains isolated from Egyptian buffalo, indicating that this gene has been associated with infection in these animals since at least 1974. We cannot be certain that the entire insert, including the prophage, was also present in these previous infections as we do not have genomic sequences from these animals. However, previous studies have suggested that toxins can be acquired through a lysogenic conversion during an infection by a β-corynephage [2,48], and this gene in the buffalo isolates is downstream from a corynephage in the same insertion sequence. If these studies are correct, then the proximity of the corynephage and the toxin gene supports the premise that the acquisition of the two together is linked in buffalo, but it is certainly not definitive proof.

Toxins can cause disease. When injected intradermally into susceptible animals, the toxin from *C*. *diptheriae* resulted in erythema, induration, and dermonecrosis [48]. Parenteral injection caused myocarditis, polyneuritis, and focal necrosis in organs that included the adrenal glands, kidneys, and liver [48]. The toxin gene described in this study was detected in *C*. *pseudotuberculosis* isolated from buffalo with OSD [16]. The presence of this toxin may be responsible for the unique disease manifestations seen in the infected buffaloes. These animals have huge abscesses in the draining lymph nodes of dewlap, belly, and limbs that can extend across the whole limb, giving an aspect of elephantiasis. Other manifestations include skin eruptions and hair loss around the eruptions, extensive dermal necrosis, spontaneous bleeding from the skin, rapid respiration, coughing, dyspnea, and haemoglobinuria [13,16]. In contrast, horses infected by *C*. *pseudotuberculosis* (biovar Equi), which lack the toxin, have abscesses in different locations that include the pectoral or ventral abdomen, or in internal organs, or ulcerative lymphangitis of the limbs [12]. As buffalo are immune to any strains from the Ovis biovar [49], and there has never been a report of buffalo infected with any other strain within the Equi biovar, a possible preventative measure could be to use an inactivated form of the *C*. *pseudotuberculosis* diphtheria toxin as an antigen in a vaccine.

**Fig 5:**
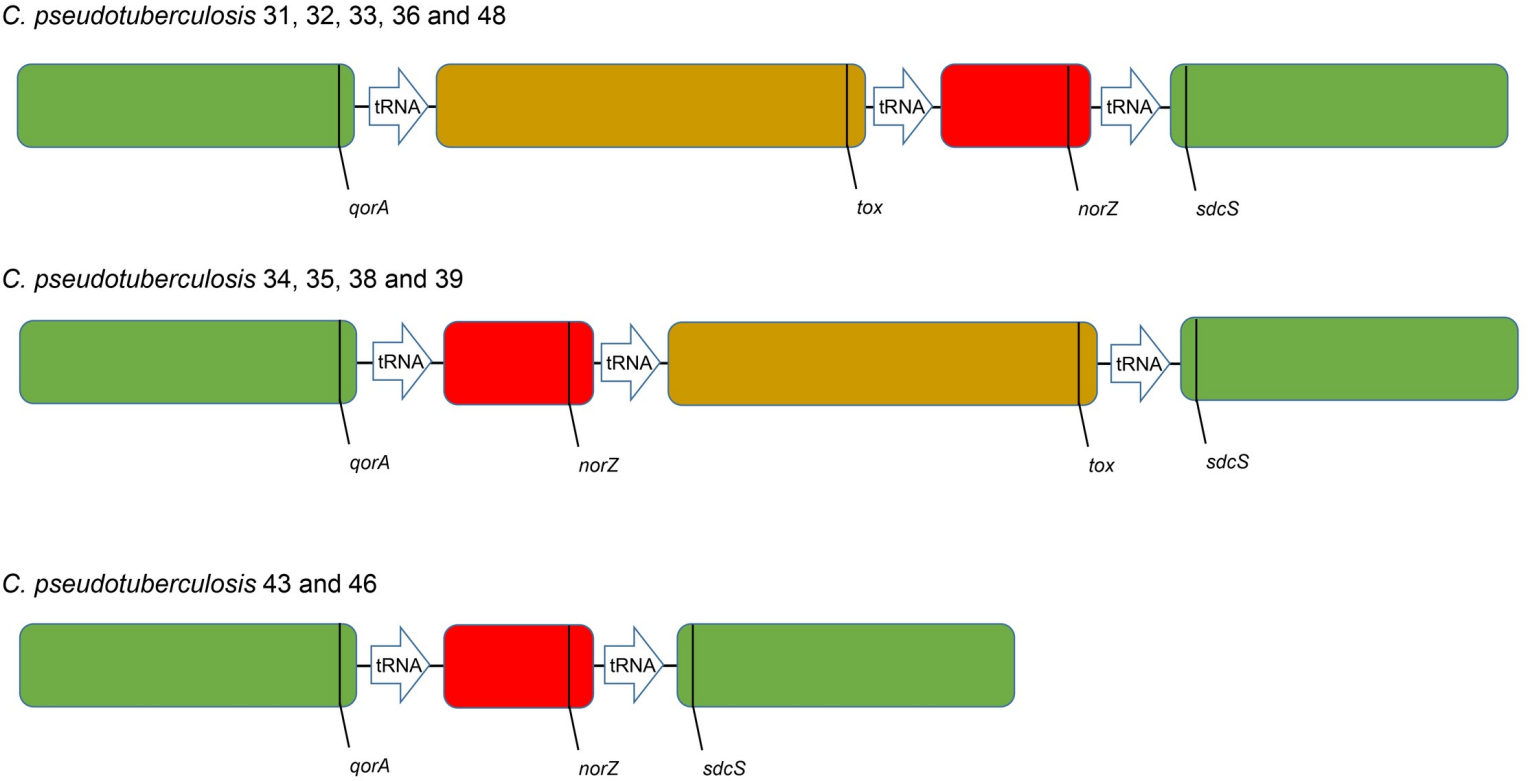
Synteny graph of 11 *Corynebacterium pseudotuberculosis* strains isolated from Egyptian buffalo.

A synteny graph including all the *C*. *pseudotuberculosis* buffalo genomes showed two large regions (green blocks) that had the same relative position, and two smaller regions (brown and red blocks) that were close to each other, but had a variable position or were missing (Fig 5). These variable regions are found within the same pathogenicity island PiCp12 and are flanked by tRNA-Arg-ACG genes. The brown block shows the 36.6 kb insertion that harbors the prophage and the diphtheria toxin (*tox*), and is absent in strains 43 and 46. The red block is also present in the genomes of the Ovis and Equi strains, and contains the Nitric-oxide reductase (*norZ*) gene. This enzyme is a defense against the cytotoxic actions of Nitrous-oxide (NO) that the host uses as a defensive mechanism [50]. As stated previously, the tRNA-Arg genes are known to be a hotspot for phage integration [45] and the fact that we see these rearrangements within genomes isolated during the same outbreak, in the same region and from the same host species confirms that this is a volatile region in these genomes. As if in confirmation of this, strains 43 and 46 were found to be positive for the toxin gene by PCR when originally isolated from infected animals. By the time they were sequenced, this region was missing, indicating that there was an excision of the 36.6 Kb insert that occurred between isolation and sequencing. Another indication of volatility is the fact that we observed not only a prophage inserted in a copy of a tRNA-Arg gene, but also saw rearrangements within this same region in other genomes that were collected during a short period of time during the summer when this disease outbreak occurred.

**Fig 6:**
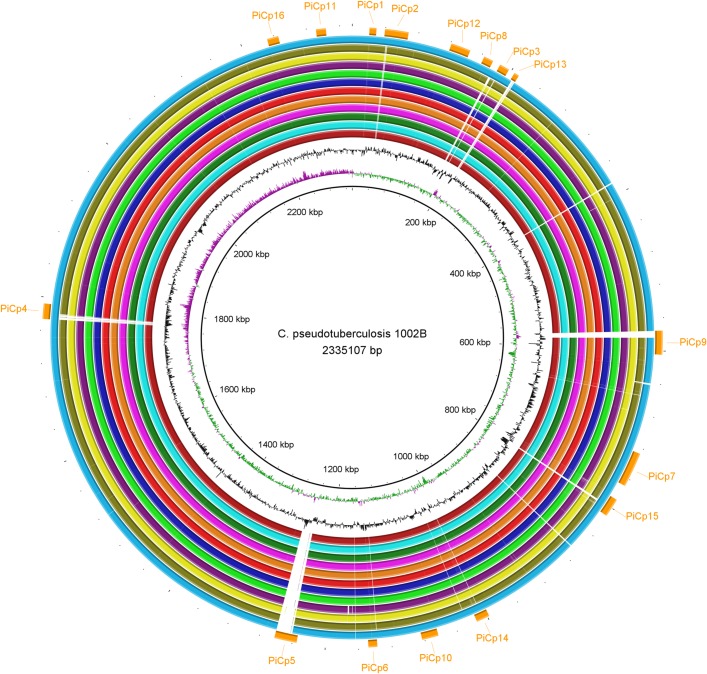
Circular genomic maps comparing *Corynebacterium pseudotuberculosis* 1002B (biovar Ovis) with other Equi strains. The rings, from the inner to outer circle, are strain 1002B, CG Skew, CG Content, strains 31, 258, E19, MB11, MB14, MB30, MB66, 316, CIP52.97, 1/06-A, Cp162 and 262, and pathogenicity islands.

**Fig 7:**
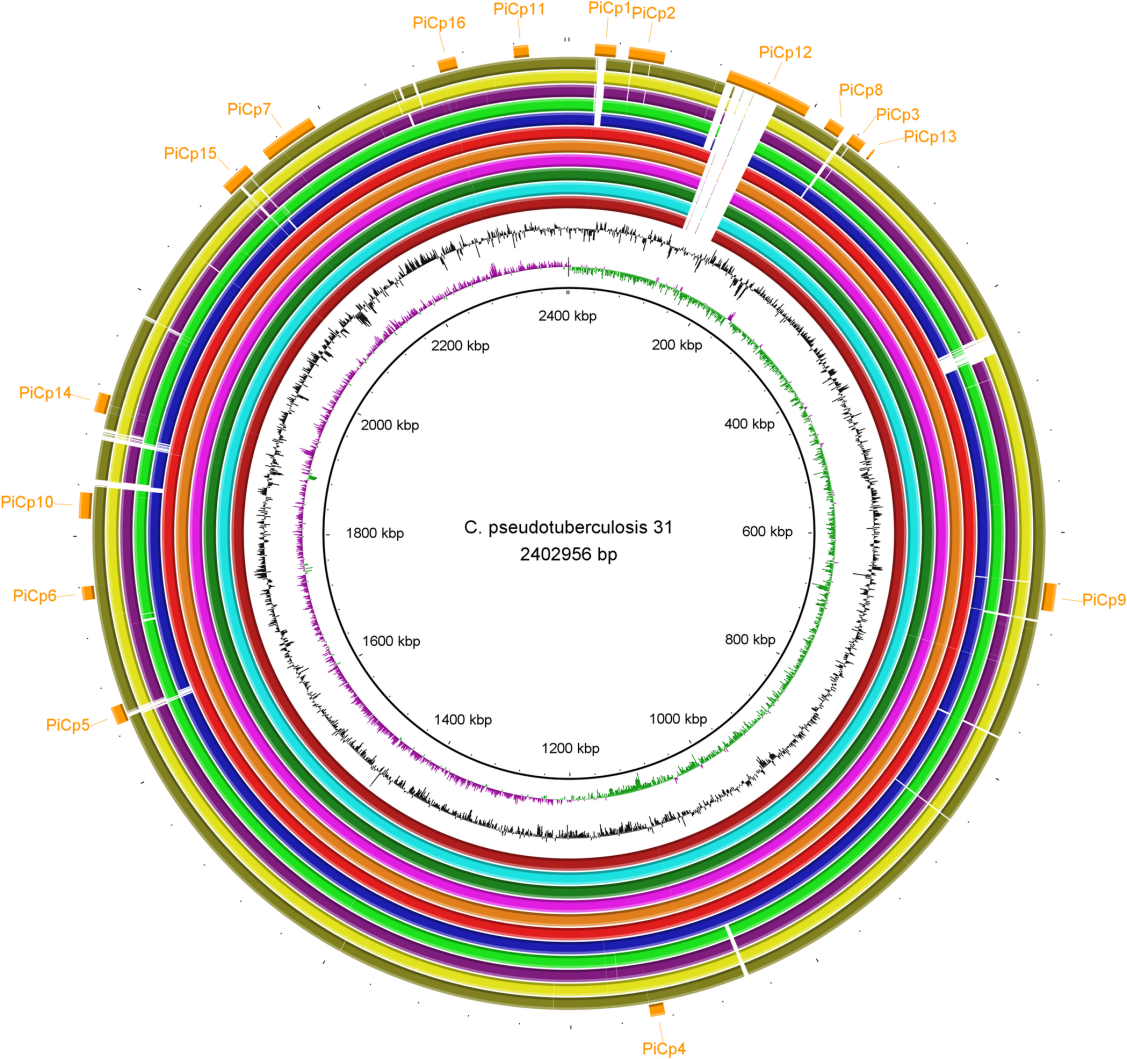
Circular genomic maps comparing *Corynebacterium pseudotuberculosis* 31 with other Equi strains. The rings, from the inner to outer circle, are strain 31, CG Skew, CG Content, strains 258, E19, MB11, MB14, MB30, MB66, 316, CIP52.97, 1/06-A, Cp162 and 262, and pathogenicity islands.

A previous study showed that all Equi strains shared the same deletion pattern when compared to strains in biovar Ovis [8]. When we compared the Equi strains with a single Ovis strain, 1002B, we, too, found that they all shared the same deletion pattern except for strain 262 (Fig 6). However, the comparison between Equi strain 31 and the other Equi strains showed that E19, 258, MB11, MB14, MB30 and MB66 had no gaps, besides the 36.6 Kb insertion exclusive of buffalo isolated strains. Also, nitrate reductase genes are missing in the strains that have gaps (Fig 7). This suggests that the gaps in those Equi strains are assembly issues instead of genetic differences.

### Phylogenomics

**Fig 8:**
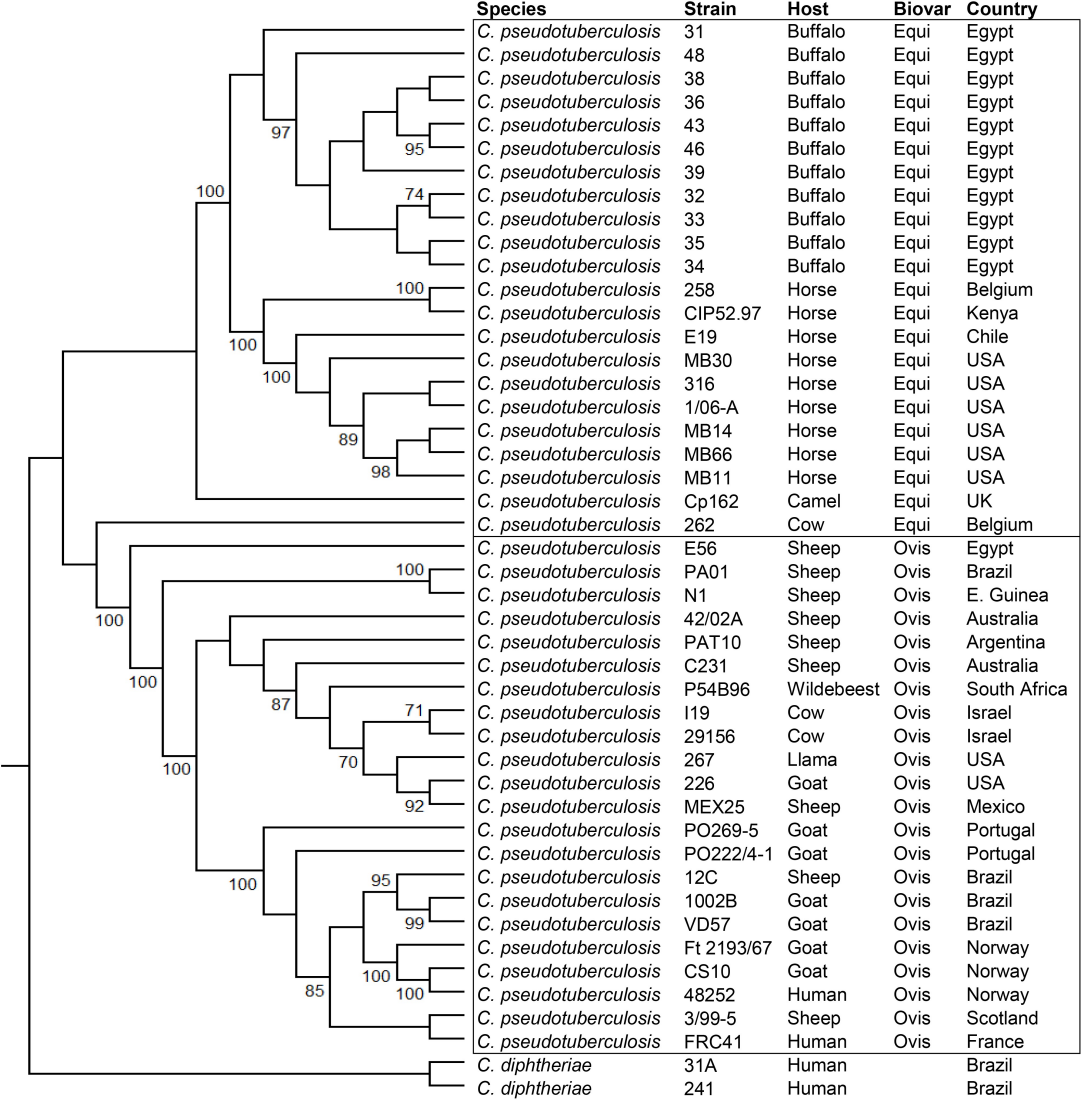
Phylogenomic tree of *Corynebacterium pseudotuberculosis* genomes based on the proteome of 44 complete genomes, generated by PEPR. The core proteins were used to produce a tree, and additional protein families were added to refine subtrees with low bootstrap values.

The phylogenomic tree generated by PEPR has two clusters representing the two biovars, with support values of 100 (Fig 8). In the Equi cluster, the buffalo were clearly separated from the horse isolates, which were collected in different countries. In the Ovis cluster, a group containing most of the goat isolates were separated from other hosts. These results suggest that *C*. *pseudotuberculosis* strains are grouped by host, at least in the Equi biovar. The fact that Equi strains 262, isolated from a cow (*Bos taurus*), and Cp162, isolated from a camel are outside of the two biovar clusters, with support values of 100, corroborates this hypothesis. Also, strain 262 was closer to biovar Ovis, as suggested by the circular map (Fig 6). It is possible that isolations from additional hosts like camels will create additional subclusters based on these hosts.

**Fig 9:**
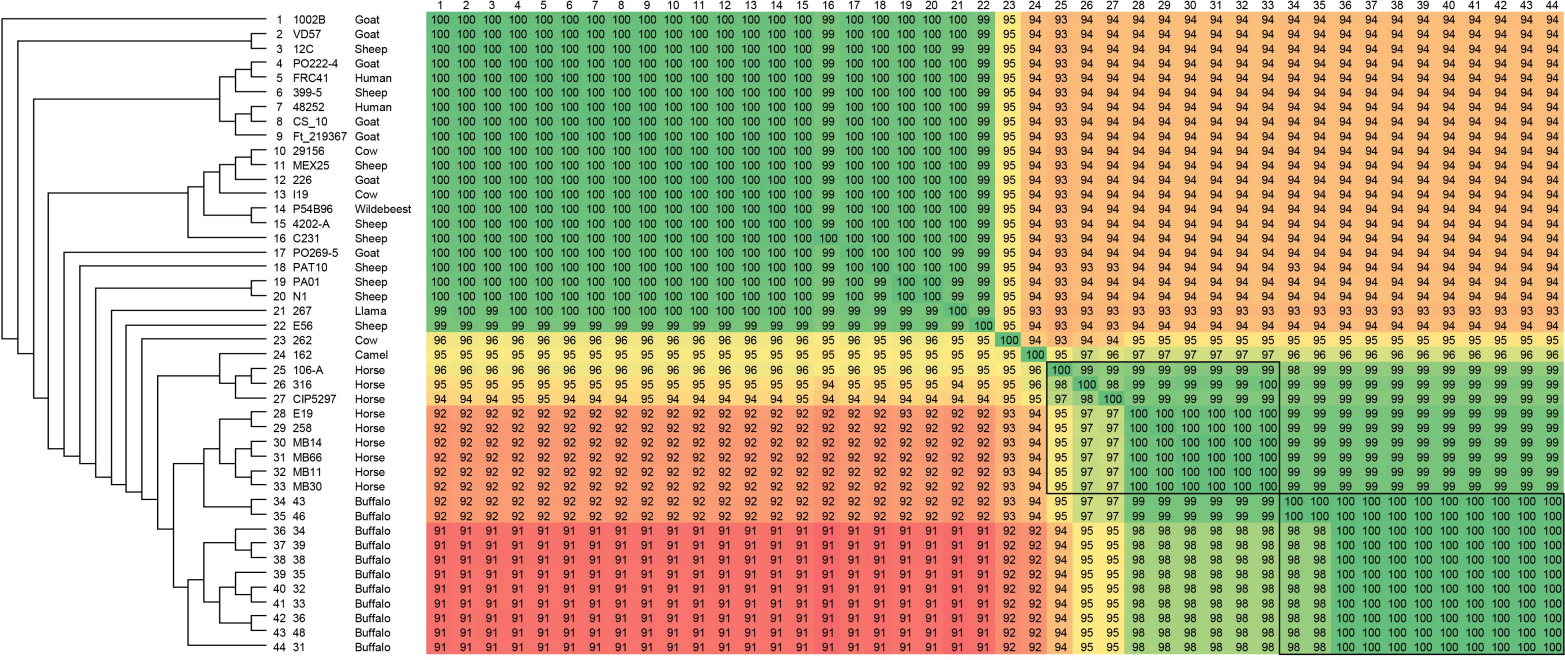
Phylogenomic tree of *Corynebacterium pseudotuberculosis* genomes based on the variable content of 44 complete genomes. The percentages of similarity were plotted on a heatmap generated by Gegenees 2.2.1, and then used to produce a phylogenomic tree using Splitstree v4.14.2 with the UPGMA method.

The phylogenomic tree produced by Gegenees showed two clusters representing the two biovars, with similarity percentages within Equi strains (92–99%) being lower than within Ovis strains (99–100%) (Fig 9), a result similar to what was found previously [8]. Here, the clustering of strains by host is also apparent. Buffalo are grouped with the horse and camel isolates, with strains 43 and 46 separated from the rest of the buffalo isolates, probably due to the absence of the 36.6 Kb insertion in PiCp12. The heatmap shows higher values of similarity within isolates of the same type of host, with 262 (the cow isolate) and 162 (the camel isolate) having the lowest values of similarity when compared to the other Equi strains.

### Pangenomics

The “pangenome” is the complete gene inventory of a species. The “core genome” is the subset of orthologous genes present in all genomes, the “accessory genome” is a subset present in more than one genome, but not all genomes. “Singletons” are genes that are present in only one genome [51]. A previous *C*. *pseudotuberculosis* study identified 1,504 genes in the core genome and a pangenome with 2,782 genes. The pangenome was characterized as open [8], meaning that sequencing new genomes should significantly contribute to the identification of new genes and thus better characterize the genetic repertoire of the species [52].

Using PATRIC’s Protein Family Sorter, we identified a pangenome that included 2,172 protein families among those genomes isolated from buffalo. Most of these same protein families were also conserved as part of the core genome, which included 2,058 families. The accessory genome was limited to 91 protein families and 13 singletons, indicating that these genomes are all very similar. Analyzing the 11 buffalo with the other 33 strains public available showed that the *C*. *pseudotuberculosis* pangenome has 3,067 genes, and 1,541 in its core genome. In addition, we used the Proteome Comparison tool, a bidirectional BLASTP analysis, to examine the genomes and found 48 genes that were unique to the buffalo isolates. All of these were part of the 36.6 kb prophage that is located in the PiCp12 island.

When *C*. *pseudotuberculosis* strain 31 was used as the reference to compare all the strains isolated from buffalo, a strong homology was shared across the genes within these genomes. All genes had a highly conserved sequence identity of at least 90.2%. Also, we identified regions absent in the Ovis biovar that were present in most of the genomes of the Equi strains (S2 Table). A comparison of the functionality of these genes was conducted to look for metabolic and functional changes across the different groups. As expected, the buffalo genomes were all consistently similar, but when compared to genomes from the Ovis biovar, we saw that the buffalo genomes had some unique functionality. The buffalo genomes contained CRISPR and phage genes that were absent in Ovis. They also included the genes involved in nitrate reduction, and additional genes that are part of molybdenum cofactor biosynthesis. The presence of nitrate reductase is one characteristic that differentiates the biovar Equi from Ovis [5,8].

**Fig 10:**
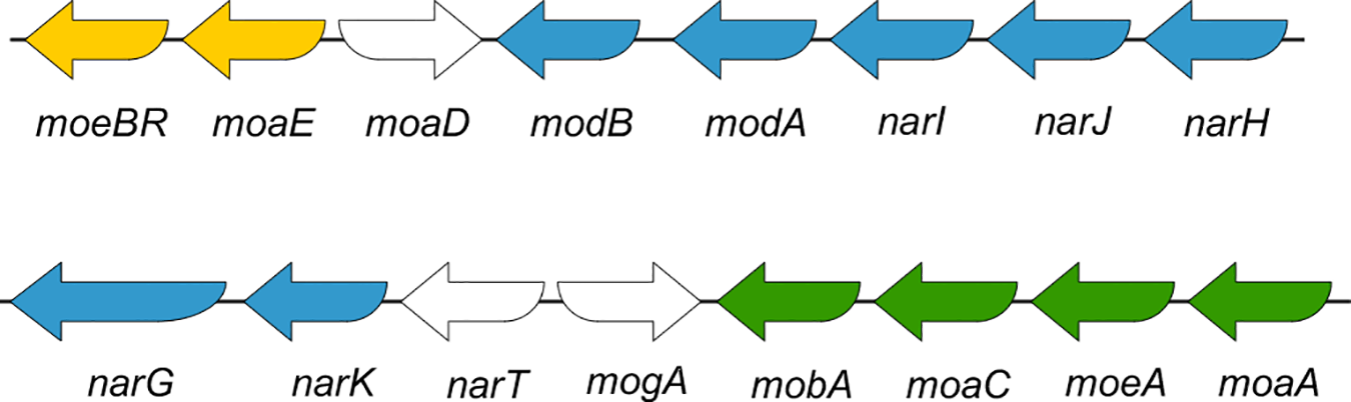
Organization of nitrate reductase and molybdenum cofactor biosynthesis genes in *Corynebacterium pseudotuberculosis* 31. Genes with the same color are transcribed in the same operon.

The use of nitrogen oxides as alternative electron acceptors, providing energy in an anaerobic environment and contributing to the persistence of the bacteria in their host, has been suggested as an advantage in adaptation to an intracellular lifestyle [50]. When we examined the region containing nitrate reduction and molybdenum cofactor biosynthesis genes, we found 13 of 16 genes were in operons: *moeBR-moaE*, *narKGHJI-modAB*, and *mobA-moaC-moeA-moaA* (Fig 10). The respiratory nitrate reductase enzyme has three subunits that are the product of the genes *narGHI*. NarI, the gamma subunit, is a transmembrane peptide that oxidizes quinol, liberates protons in the periplasm, and transports the electrons to NarH. NarH is the beta subunit, and it transports electrons from NarI to NarG. The alpha subunit NarG contains the catalytic domain that requires a molybdenum cofactor Mo-(bis-MGD) for activity. This subunit uses nitrate as the electron acceptor, reducing it to nitrite [53]. NarJ assists in the insertion of molybdenum cofactor in NarG, which must happen before the interaction between NarGH and NarI [54]. NarK is a transporter that performs nitrate/nitrite exchange [55]. NarT is probably involved in nitrate/nitrite transport as it has 80% identity with *narK*, and also includes the same Major Facilitator Superfamily (MFS) domain. MFS transporters are single-polypeptide secondary carriers that transport small solutes in response to a chemiosmotic ion gradients [56]. The nitrate reductase has been suggested as a drug target in *Mycobacterium tuberculosis* [53], and could be used in a similar fashion in *C*. *pseudotuberculosis*.

Several studies have attempted to define the roles that the *nar* genes play in survival within the host. NarGH nitrate reductase provides resistance from acid stress and reactive nitrogen species in mycobacteria [57]. A *narG* mutant of *M*. *tuberculosis* was unable to persist in the lung, kidney and liver of immunocompetent mice, but it was able to grow and persist in the spleen as the wild-type strain, suggesting that the role of nitrate reduction in virulence is tissue specific [58]. However, another study showed that there no difference in persistence in the mice lungs between a *narG* mutant and wild-type *M*. *tuberculosis*, probably because mouse granulomas are not sufficiently hypoxic to affect the growth and survival of these bacteria [59]. The benefit, if any, that these genes give to the Equi strains has not been determined, but could be a target of further experimentation to see if they are important in either host specificity or disease manifestation. A genetic manipulation of *narG* in *C*. *pseudotuberculosis* could shed light on the different disease manifestations seen between hosts infected with either of the two biovars.

NarG requires molybdenum to function, and, in the buffalo genomes, the molybdenum transport genes are adjacent to the ones needed for nitrate reduction. The molybdenum cofactor biosynthesis operon *modABC* encodes a molybdate transmembrane transporter. ModA binds molybdate, ModB is a transmembrane subunit, and ModC provides the energy required for transportation by ATPase activity [60]. MoaA and MoaC convert a guanine nucleotide (GTP) to cyclic pyranopterin monophosphate (cPMP). MoaD and MoaE are subunits of the molybdopterin (MPT) synthase, which converts cPMP to MPT. MoeBR (a sulfurtransferase) activates MPT synthase by transferring sulfur groups to MoaD subunit. MogA (an adenylyltransferase) adenylates MPT, resulting in MPT-AMP. Molybdate is added by MoeA (MPT Mo-transferase), resulting in Mo-MPT (molybdenum cofactor). MobA (guanylyltransferase) converts Mo-MPT to a bis-Mo-MPT intermediary and attaches two guanines (GMP) to its phosphate groups, converting it to bis-MGD (molybdopterin guanine dinucleotide), which is the required cofactor for dimethylsulfoxide reductase (DMSO) enzymes, including nitrate reductase [60,61]. The insertion of the molybdenum cofactor in nitrate reductase NarGHI is performed by NarJ [62]. We found that the genes coding the transmembrane and ATP binding subunits of molybdate transporter (*modB* and *modC*) are fused in *C*. *pseudotuberculosis* and are transcribed with the nitrate reductase genes *narKGHJI* in the same operon, as predicted by FgenesB. The fusion between *modB* and *modC* does not appear to affect the phenotype, as all Equi strains that have been sequenced are positive for nitrate reduction, and they all share this fusion.

### Specialty genes search

Specific genes that function as virulence factors can determine bacterial adhesion, invasion, colonization, dissemination within the host and evasion of the immune system [63]. The diphtheria toxin gene was the only virulence factor found that was unique to the buffalo isolates. An additional virulence factor, inositol-1-phosphate synthase, a product of the *ino1* gene, was found in the buffalo strains. This gene is broadly shared across the *Corynebacterium*. Ino1 catalyzes the first step in the synthesis of inositol [64], a compound required for the production of essential cell wall lipoglycans in *Mycobacterium* [65], and is a major thiol that plays a role in protection from oxidative stress [66,67]. In an experiment using *M*. *tuberculosis*, the role that *ino1* plays in virulence was demonstrated when the CFUs of *ino1* mutants fell sharply, and the bacteria were virtually cleared in seven days by infected macrophages, while they remained constant in wild type strains [68]. Furthermore, mice infected with wild type *ino1* strains died in 38 days, while mice infected by the *ino1* mutant were alive and healthy when the experiment concluded at 56 days post infection [68]. The role, if any, that this gene plays in the virulence of *C*. *pseudotuberculosis* has not yet been determined.

Another virulence factor identified was the exotoxin phospholipase D (*pld* gene), which promotes bacterial dissemination by degradation of sphingomyelin in endothelial cell membranes, and also plays a role in macrophage death [69,70]. In strain 31, *pld* was found to have a frameshift mutation near the 3’, and it was suggested that this could decrease the ability of this strain to spread throughout the host, and this, along with the *tox* gene, have been proposed as either a requirement for infection or a geographic variant [8]. This mutation was not seen after resequencing strain 31, however, nor did we find it any other genomes used in this study. This suggests that the original finding was probably a sequencing artifact.

Pili are structures responsible for bacterial adhesion and play a major role in the initiation of extracellular and intracellular invasion and proliferation [71]. Pathogenicity islands PiCp7 and PiCp15 harbor the pilus gene cluster *spaA* (*srtB-spaA-srtA-spaB-spaX-spaC*) and *spaD* (*srtC-spaD-spaY-spaE-spaF*), respectively [8]. The genes *srtABC* are specific pilus sortases, while *spaAD*, *spaBE* and *spaCF* are major, base, and tip proteins, respectively. The genes *spaX* and *spaY* have unknown functions [72]. Specific sortases cleave the LPTxG motif of the pilin proteins and polymerize them to assembly the pilus, while the housekeeping sortase incorporates the final structure to the cell wall [73]. When we compared the Ovis and Equi strains, we saw some unique differences in these regions.

**Fig 11:**
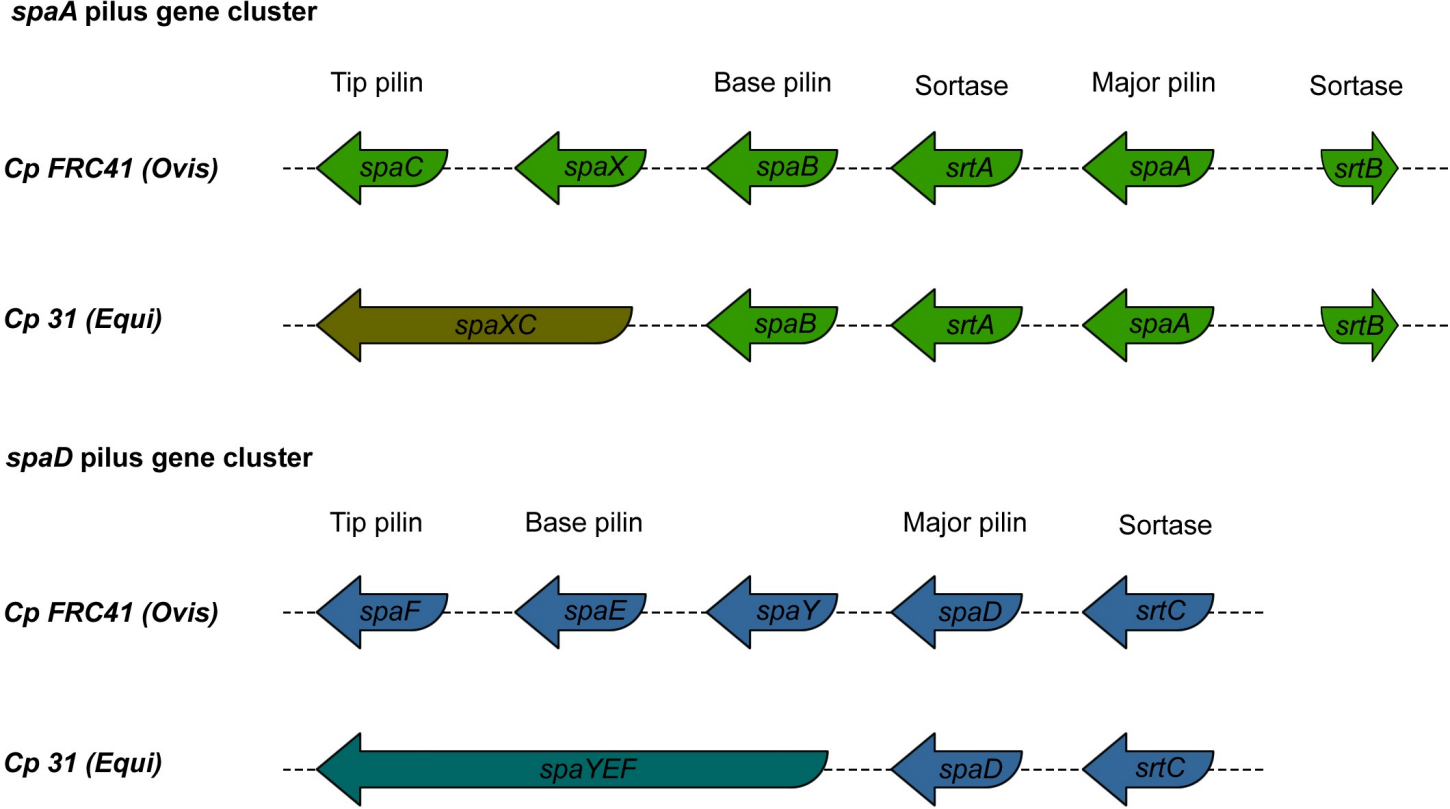
Comparison of pilus gene cluster *spaA* and *spaD* in *Corynebacterium pseudotuberculosis* isolated from buffalo, represented by strain 31, and biovar Ovis, represented by strain FRC41.

A comparison of pilus gene clusters *spaD* and *spaA* showed conservation among buffalo isolates (Fig 11) and polymorphisms when compared to other Equi strains. The *spaD* pilus genes from the buffalo isolates had a fusion between three genes, including the base and tip pilin (genes *spaE-spaF-spaY*), which is also seen in the Equi strains 258, E19 (isolated from a horse) and Cp162 (camel isolate). In all of the buffalo isolates except for strain 31, the major pilin gene *spaA* has a frameshift. A similar frameshift is also seen in all the other Equi isolates except for strains 316 (isolated from a horse), and 262 (isolated from a cow). Fusion is also seen between with the *spaC* (tip pilin) and *spaX* genes in the buffalo isolates. Not all members of Equi share this fusion, but it is also found in 262 (cow), Cp162 (camel), E19 and 258 (horse isolates). The major pilin (*spaA*) gene is frameshifted in most of the Equi strains.

In *C*. *diphtheria*, the three pili structures SpaA, SpaD and SpaH have been found to be necessary for adhesion to pharyngeal, laryngeal and lung human epithelial cells, respectively. In this same species, a deletion of the major pilin gene showed that the tip and base pilin were the most important proteins for adhesion on the host cell wall [74]. The frameshift in *spaA* (major pilin) found in *C*. *pseudotuberculosis* may be not critical to the adhesion. Although, given the importance of base and tip pilin, the fusions of these genes and the conservation within strains may represent an adaptation to the buffalo host, or to mutation events in the immediate ancestor this particular outbreak. More data, perhaps from isolates of future outbreaks of OSD before could determine if this an anomaly or a true adaptation. The polymorphisms in the Equi pilus genes could be due to the higher variability of host species in this biovar, leading to specificity for tissues of different host species [74].

The cow isolate 262 is classified as Equi and has the same nitrate reductase genes, but the sequences of two pilus genes (*srtB* and *spaA*) are identical to those found in the Ovis biovar. Also, the phylogenetic tree (Fig 8) and the circular map (Fig 7) showed the similarity of this strain with the Ovis isolates, yet it could be viewed as ancestral to that clade. Perhaps other Equi isolates, including those from *B*. *taurus* cattle, will help determine if this is an anomaly, or perhaps the first of a new biovar of *C*. *pseudotuberculosis*.

## Conclusion

*C*. *pseudotuberculosis* strains isolated from buffalo showed an overall synteny, conservation in pilus genes, and a unique insertion that contained both a corynephage and the diphtheria toxin gene. This insertion could explain the expansion of the known host range for *C*. *pseudotuberculosis* to include buffalo, as these genes may play a role in adaptation to this new host. The tRNA-Arg gene was identified as a hotspot of phage insertion and rearrangements, events observed during in this outbreak of OSD. By comparison with Ovis strains, we identified and described the known nitrate reductase (*narGHI*) genes and identified genes involved molybdenum cofactor biosynthesis, which are necessary for the action of the *nar* genes. A phylogenomic tree confirmed a clear separation between the Ovis and Equi biovars, and indicated that Equi strains are clustered depending on the host they infected.

## Supporting information

S1 Fig Comparison of three versions of *Corynebacterium pseudotuberculosis* 31 genome assembly and strain 32. The rings, from the inner to outer circle, are strain 31 v3 (CP003421.3), GC skew, GC content, strains 31 v1 (CP003421.1), 31 v2 (CP003421.2), and 32 (CP015183.1), and pathogenicity islands.
(TIF)

S1 File Differences in the assembly versions of *Corynebacterium pseudotuberculosis* strain 31. (DOCX)

S1 Table Gene content in the three genome sequences present in the first version of *Corynebacterium pseudotuberculosis* 31 (CP003421.1) and absent in the second version (CP003421.2). (DOCX)

S2 Table Accession number of the prophage sequences in *Corynebacterium pseudotuberculosis* strains isolated from buffalo. (DOCX)
