# Supplementary figures and images for: Comparative genomic analysis between Corynebacterium pseudotuberculosis strains isolated from buffalo
Source: PLoS One. 2017 Apr 26;12(4):e0176347. doi: 10.1371/journal.pone.0176347 (PMC5406005; doi:10.1371/journal.pone.0176347)

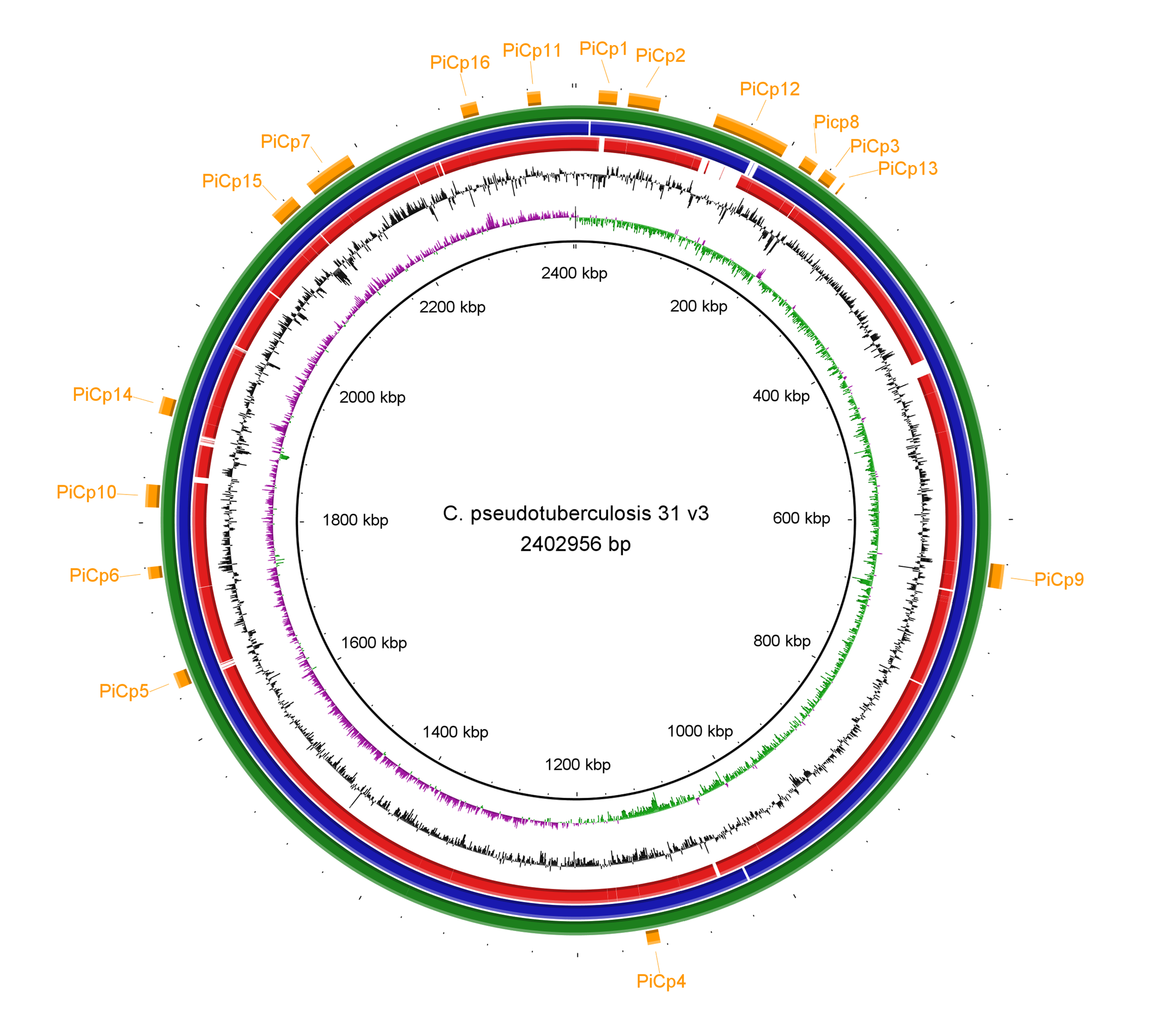

Supplement: S1 Fig — The rings, from the inner to outer circle, are strain 31 v3 (CP003421.3), GC skew, GC content, strains 31 v1 (CP003421.1), 31 v2 (CP003421.2), and 32 (CP015183.1), and pathogenicity islands. (TIF) [file pone.0176347.s001.tif]
