# Supplementary material for: Comparative genomic analysis between Corynebacterium pseudotuberculosis strains isolated from buffalo
Source: PLoS One. 2017 Apr 26;12(4):e0176347. doi: 10.1371/journal.pone.0176347 (PMC5406005; doi:10.1371/journal.pone.0176347)
Supplement: S1 File — (DOCX) [file pone.0176347.s002.docx]

**Supporting Information**

**S1 File. Differences in the assembly versions of *Corynebacterium pseudotuberculosis* strain 31.**

Clockwise, the first gap in strain 31 v2 (CP003421.2, S1 Fig) corresponds to a 1.95 kb sequence containing two CDSs of the first PAI (PiCp1). The second gap, which is 8.9 Kb corresponds to red block in Figure 3’s synteny graph (Fig 3), includes the diphtheria toxin gene and is flanked by tRNA-Arg-ACG genes. The third gap corresponds to a 2.3 Kb sequence that contains two, flanked by tRNA-Glu genes. All of these three regions were present in the previous assembly version of this genome (S1 Table).
