# Supplementary material for: Comparative genomic analysis between Corynebacterium pseudotuberculosis strains isolated from buffalo
Source: PLoS One. 2017 Apr 26;12(4):e0176347. doi: 10.1371/journal.pone.0176347 (PMC5406005; doi:10.1371/journal.pone.0176347)
Supplement: S1 Table — (DOCX) [file pone.0176347.s003.docx]

**S1 Table. Gene content in the three genome sequences present in the first version of *Corynebacterium pseudotuberculosis* 31 (CP003421.1) and absent in the second version (CP003421.2).**

| **AA** | **RefSeq locus tag** | **Gene** | **Product** | **Gap size** |
| --- | --- | --- | --- | --- |
| 234 | Cp31_0012 |  | Hypothetical protein | 1.95 kb |
| 171 | Cp31_0013 |  | Hypothetical protein |  |
| 39 |  |  | Hypothetical protein | 8.9 kb |
| 487 | Cp31_0136 | *sdcS* | Putative membrane protein |  |
| 282 | Cp31_0137 | *glcT* | Beta-glucoside bgl operon antiterminator, BglG family |  |
| 526 | Cp31_0138 | *nagE* | PTS system, N-acetylglucosamine-specific IIA component / PTS system, N-acetylglucosamine-specific IIB component (EC 2.7.1.69) / PTS system, N-acetylglucosamine-specific IIC component |  |
| 140 | Cp31_0139 |  | COG1263: Phosphotransferase system IIC components, glucose/maltose/N-acetylglucosamine-specific |  |
| 265 | Cp31_0140 |  | Hypothetical protein |  |
| 750 | Cp31_0141 | *norZ* | Nitric-oxide reductase (EC 1.7.99.7), quinol-dependent |  |
| 39 |  |  | Hypothetical protein | 2.3 kb |
| 469 | Cp31_0915 |  | Hypothetical protein |  |
