# Supplementary material for: Comparative genomic analysis between Corynebacterium pseudotuberculosis strains isolated from buffalo
Source: PLoS One. 2017 Apr 26;12(4):e0176347. doi: 10.1371/journal.pone.0176347 (PMC5406005; doi:10.1371/journal.pone.0176347)
Supplement: S2 Table — (DOCX) [file pone.0176347.s004.docx]

**S2 Table. Accession number of the prophage sequences in *Corynebacterium pseudotuberculosis* strains isolated from buffalo.**

| **Species** | **Strain** | **Prophage** | **Accession number** |
| --- | --- | --- | --- |
| *C. pseudotuberculosis* | 31 | LGCM-V1 | KY566218 |
| *C. pseudotuberculosis* | 32 | LGCM-V2 | KY613597 |
| *C. pseudotuberculosis* | 33 | LGCM-V3 | KY624610 |
| *C. pseudotuberculosis* | 34 | LGCM-V4 | KY624611 |
| *C. pseudotuberculosis* | 35 | LGCM-V6 | KY624612 |
| *C. pseudotuberculosis* | 36 | LGCM-V5 | KY624613 |
| *C. pseudotuberculosis* | 38 | LGCM-V7 | KY624614 |
| *C. pseudotuberculosis* | 39 | LGCM-V8 | KY624615 |
| *C. pseudotuberculosis* | 48 | LGCM-V9 | KY624616 |
